# AI pitfalls and what not to do: mitigating bias in AI

**DOI:** 10.1259/bjr.20230023

**Published:** 2023-09-20

**Authors:** Judy Wawira Gichoya, Kaesha Thomas, Leo Anthony Celi, Nabile Safdar, Imon Banerjee, John D Banja, Laleh Seyyed-Kalantari, Hari Trivedi, Saptarshi Purkayastha

**Affiliations:** 1 Department of Radiology, Emory University, Atlanta, United States; 2 Laboratory for Computational Physiology, Massachusetts Institute of Technology, Cambridge, Massachusetts, United States; 3 Division of Pulmonary, Critical Care and Sleep Medicine, Beth Israel Deaconess Medical Center, Boston, Massachusetts, United States; 4 Department of Biostatistics, Harvard T.H. Chan School of Public Health, Boston, Massachusetts, United States; 5 School of Computing, Informatics, and Decision Systems Engineering, Arizona State University, Tempe, United States; 6 Emory University Center for Ethics, Emory University, Atlanta, United States; 7 Department of Electrical Engineering and Computer Science, Lassonde School of Engineering, York University, North York, United States; 8 School of Informatics and Computing, Indiana University Purdue University, Indianapolis, United States

## Abstract

Various forms of artificial intelligence (AI) applications are being deployed and used in many healthcare systems. As the use of these applications increases, we are learning the failures of these models and how they can perpetuate bias. With these new lessons, we need to prioritize bias evaluation and mitigation for radiology applications; all the while not ignoring the impact of changes in the larger enterprise AI deployment which may have downstream impact on performance of AI models. In this paper, we provide an updated review of known pitfalls causing AI bias and discuss strategies for mitigating these biases within the context of AI deployment in the larger healthcare enterprise. We describe these pitfalls by framing them in the larger AI lifecycle from problem definition, data set selection and curation, model training and deployment emphasizing that bias exists across a spectrum and is a sequela of a combination of both human and machine factors.

## Introduction

Despite the promise and hope of artificial intelligence (AI) to improve patient care, several real-world failures of AI systems have been documented. In addition to observing overall performance decline secondary to differences in data distribution after model deployment, subgroup evaluations have shown varying levels of performance with poor performance especially for historically underserved patients.^1,2^ These are individuals who have and continue to experience systematic and persistent barriers to accessing quality healthcare due to various factors, including race, ethnicity, socioeconomic status, geography, and language barriers. Bias in medical research is defined as the intentional or unintentional introduction of systematic error into sampling or testing by, intentionally or not, selecting or encouraging one outcome or answer over others.^3^ Multiple publications exist elaborating on the types of (largely statistical) bias, and more importantly, how to mitigate their influence on the study’s conclusions.^4–6^ Recently, with the introduction of AI and machine learning, there has been a resurgence of interest in (once again, largely statistical) bias identification^7,8^ and tools to aid in its mitigation,^9^ particularly as we move towards the real-world implementation of AI systems in various healthcare settings.

Efforts to mitigate these biases are challenging due to—lack of a unified definition of bias^10^; focus on a statistical definition of bias and a technocentric definition of bias without involving patients and communities; and *post-hoc* reflection on bias rather than as a deliverable by design.^11^ Bias audits for models tend to be piecemeal—typically performed only during model development and validation; health equity considerations should commence during data collection and curation through post-deployment monitoring.^12^ The failure and subsequent withdrawal of the Epic (^TM^) sepsis model used by 180 customers representing hundreds of hospitals provides a template to study AI failure across a spectrum.^13,14^ Upon testing at Michigan Medicine, the algorithm only identified 7% of patients whose sepsis diagnosis was missed by a clinician, failed to flag 67% of patients with sepsis despite generating alerts on 18% of all hospitalized patients resulting in alert fatigue. Before the subsequent model overhaul as a result of the publication (rather than some planned post-deployment evaluation and monitoring), it was observed that one input variable to the model was antibiotic orders by a provider—a type of data leakage since an infection is already being considered at that point. Many other lessons are noted from the sepsis use case including bypassing of regulatory oversight (due to packaging of the algorithm as a non-device decision-support tool), lack of calibration to various population differences and hospital-specific practice patterns, lack of access to the proprietary algorithm for inspection of errors and biases, and lack of process for real-world evaluation post-model deployment. In cases where remote clinicians were deployed to monitor the algorithm outputs, the constant interruption (up to 18% of hospitalized patients generated an alert) led the floor nurses to cover the video camera to limit workflow disruption.^13,14^ A similar result is summarized in a systematic review of COVID-19 prediction models on CT scans and Chest X-rays (CXRs) that found limited clinical utility due to methodological flaws and underlying biases stemming from small training data sets, data set variability, and limited integration of non-imaging data, among others. The methodological flaws likely is a reflection of the lack of diversity in the development team to allow clinicians, data analysts and patient advocacy groups to work side-by-side throughout the AI life cycle.^15^

**Figure 1.**
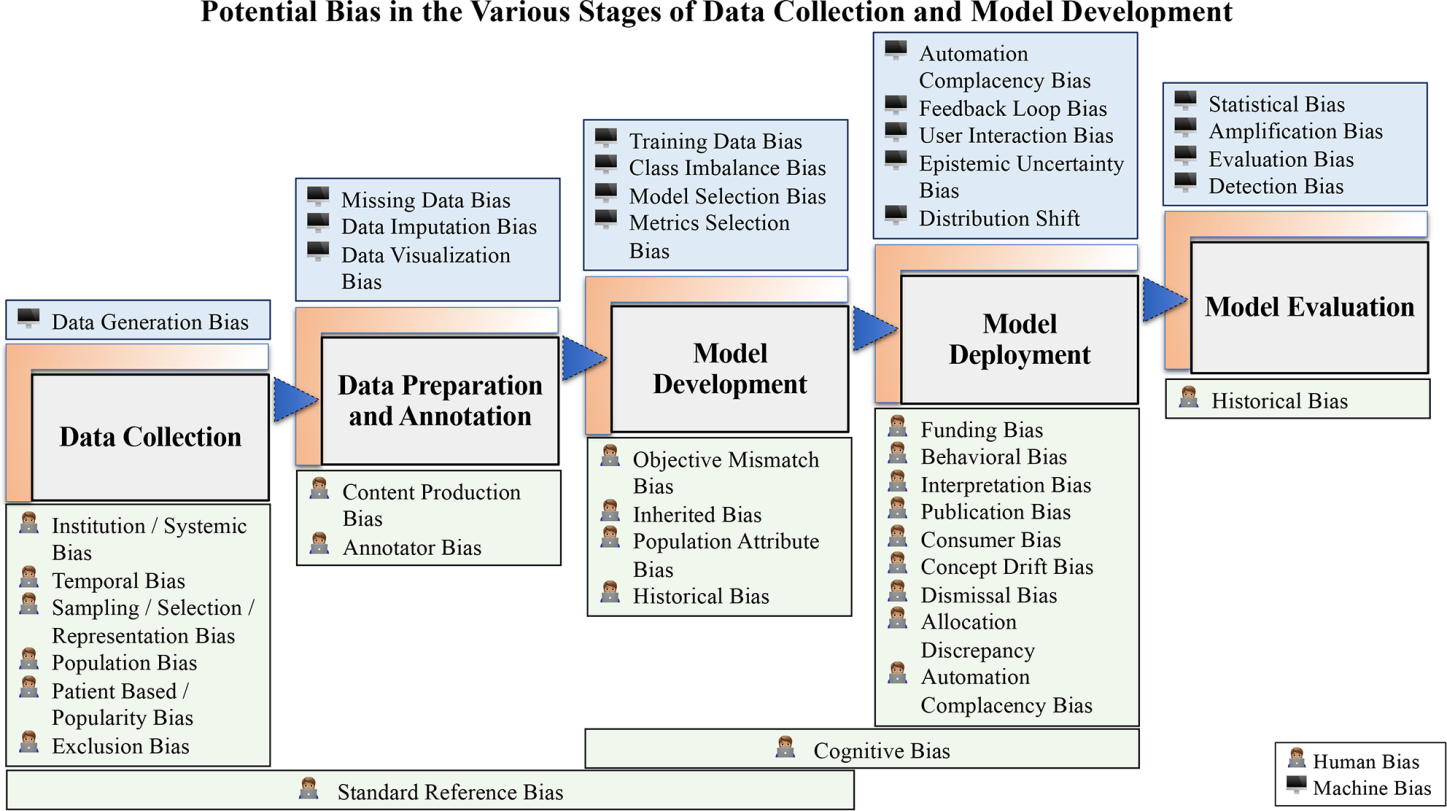
Summarizes possible biases at every stage of AI development from model development, including demonstration of the intersection of human and machine in causing bias. AI, artificial intelligence.

Imaging is at the center of inpatient and outpatient care delivery, touching a vast majority of patients. Prior articles have looked at bias in radiology images in isolation of the larger healthcare delivery system. In this paper, we provide an updated review of known pitfalls causing AI bias and discuss strategies for mitigating these biases within the context of AI deployment in the larger healthcare enterprise. We describe these pitfalls by framing them in the AI development and deployment lifecycle from problem definition, data set selection and curation, model training and deployment, and post-deployment evaluation and monitoring (Figure 1). We emphasize that bias permeates every step of the lifecycle and is a sequela of human, machine, and systems factors.

### AI pitfalls when defining a task

Bias principles are largely considered too late, if at all, when designing AI. Health outcome prediction may be biased even when attributes that are associated with suboptimal care are hidden from a neural network agent. For example, Obermeyer et al demonstrated racial bias in a commercial prediction algorithm already in use for hundreds of million ambulatory patients. The algorithm was supposed to identify patients who may benefit from referral to a case management team based on their likelihood to develop complication. The algorithm referred less Black patients with a similar disease burden compared to White patients.^1^ The algorithm developers in this case did not use a race variable in their model input to avoid bias. However, by using healthcare costs as proxy for the need for complex care, the model learned from a system where Black patients are less likely to seek and/or receive care compared to matched White patients.^1^

A similar example is an algorithm used to predict hospital length of stay to consult case managers for patients with shorter predicted length of stay to maximize throughput.^16^ The algorithm (which was abandoned before deployment) learnt that patients from less affluent zip codes are likely to have longer hospital stays and may not benefit from case management facilitation of early discharge. AI developers must always consider whether the proxy of a target outcome they are predicting or optimizing may be reflective of a systemic bias. Examples of such metrics in addition to healthcare cost include outputs of decision-making such readmission^17^ or patient no-show. Social scientists, in particular, can help explain systemic bias that may be embedded in the metric chosen. When defining a research problem or task in the context of AI, the choice of a bias-proof feature or outcome with a precise consensus definition across various stakeholders is critical. The need for cognitive diversity within the AI development team cannot be emphasized enough.

### Pitfalls in data acquisition and collection

Bias in data is one of the major source of biased algorithmic outcome.^18,19^ Comprehensive AI data sets are limited and expensive to curate. Problems in data set development include limited patient diversity, limited resolution and missing clinical confounders, limited or suboptimal quality annotations, and lack of data standards and best practice to ensure quality and reproducibility. Existing AI data sets are acquired from limited geographical regions; most lack information necessary to evaluate subgroup performance of models. Differences in image acquisition and processing may result in “shortcuts” where models take cues from non-clinical features. For example, COVID-19 prediction models have been demonstrated to learn which institution the images were obtained from rather than features pertaining to the underlying disease.^15^ Zech et al demonstrated a similar finding where models predicted pneumonia by learning where the CXRs was obtained from.^20^ To overcome this confounding, progress must be made to improve model training, evaluation, and explainability.^21^

A review of published AI articles between 2015 and 2019 in six disciplines found that data used for imaging AI models largely come from three states—California, Massachusetts, and New York—with none from 34 states.^22^ While there have been initiatives to build data sets from “data deserts”, presently, AI models are trained on data collected from tertiary care academic institutions. A review of 23 CXR data sets found that although the majority reported age and sex, only 8.7% reported race or ethnicity and 4.3% reported insurance status.^23^ Even large repositories like the UK Biobank that represents a prospective cohort of 500,000 patients are limited for some patient groups: only 6% of the cohort are of non-European ancestry.^24^ Presently, it would be difficult to further evaluate model bias on these data sets. In fact, only 8% of the UK Biobank has been used for research, with most of this research focused on patients of European ancestry. It is important to note that in some countries collection of information such as race and ethnicity are prohibited.^25,26^

Recent studies have shown that AI models can learn features that are “invisible” to a human expert. For example, CXRs can be used to predict ICD-10 codes related to cardiac disease (including conduction abnormalities) with accuracies of above 0.76.^27^ More concerning about these models is that information on social determinants of health like area deprivation index are learned and encoded on the CXRs. CXRs can also be used to predict demographic information—including insurance, self-reported race, and age.^28,29^ CXRs have also been used to predict biological age, cardiac disease outcomes and healthcare cost at 1, 3 and 5 years.^30^ Despite the surprising performance of AI models to detect these signals, there are limitations in model explainability. Why is this concerning? The “race signal” (or societal standing in general) in images may be used to inform clinical predictions and optimizations especially in this era of multimodal modeling.^31^ The fact that these models demonstrate this superior ability even when the images are modified in such a way that they are barely perceptible as medical images implies that mitigating bias will be difficult at best.

These “hidden signals” in the context of ongoing demonstrations of bias beyond imaging presents a challenge because biases exist along the AI lifecycle even before the modeling step. For example, bias exist in who gets specific types of imaging, and when. Black, Hispanic, and non-White patients are less likely to undergo diagnostic imaging in the emergency department.^32^ Inappropriate use among historically underserved patients has been demonstrated in other areas of diagnostic imaging.^33^ An investigation of bystander CPR response for witnessed cardiac arrest found that Black and Hispanic patients or females are less likely to get CPR regardless of income level or neighborhood where the cardiac arrest occurred.^34^ This means that these patients would be excluded from an imaging cohort evaluating cardiac disease outcomes. Bias in clinical parameters such as glomerular filtration rate, pulse oximetry (with undiagnosed hidden hypoxemia for historically underserved patients),^35^ differences in ICU severity scoring^36^ shape radiology data sets for machine learning through a cascade effect as they affect ordering patterns for medical images. Recent NIH funding through AIM-AHEAD^37^ and Bridge2AI^38^ programs identifies this as the **most challenging issue** for mitigating bias in available data sets; in addition to a lack of diversity in the research terms and the absence of experts to provide guidance on limitations of the data. Some groups have suggested datasheets^39,40^—a checklist to guide model developers on how to use data—to address this problem.

**Figure 2.**
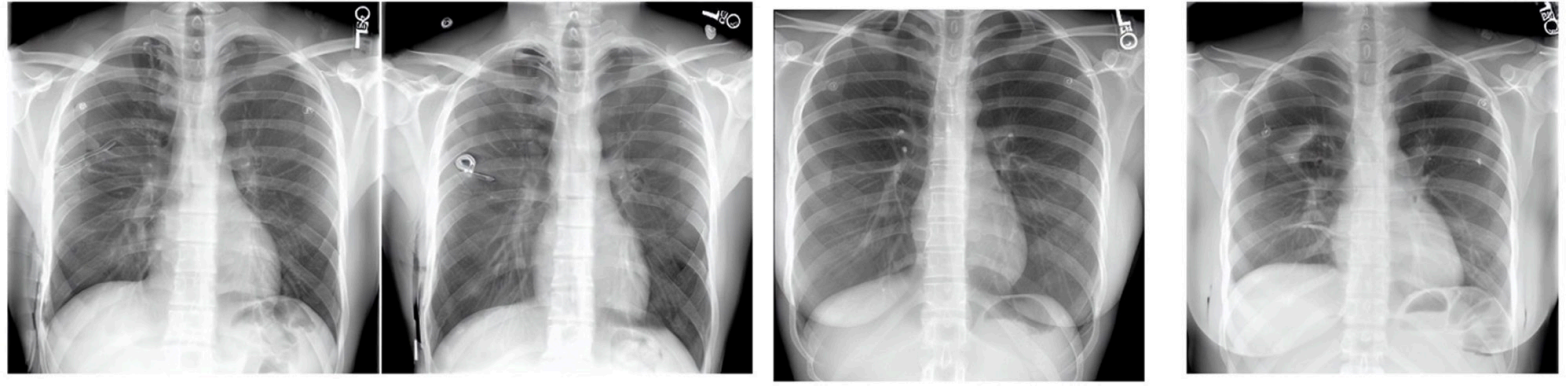
The output of Roentgen with the left two images representing “pneumothorax with chest tubes” and the right two images “pneumothorax without chest tubes”. Visual inspection shows fragmented chest drains and no obvious pneumothorax on the images without chest tubes.

Recently, many strategies have been employed to improve data availability for AI in medical imaging. Generative adversarial networks and latent diffusion models have been used to create synthetic data sets like brain MRI based on age, sex, and brain structure volumes.^41^ Our preliminary work found that signals that identify race are also contained on synthetic data sets. Theoretically, it may be easier to validate models using these controlled data sets (since one can specify how many patients to include in specific subgroups) but more work is needed to validate this. Similarly, foundation models which generate specific text-image combinations are increasingly applied to radiology. Using Roentgen,^42^ a vision-language foundation model for CXR, we generated pictures of pneumothorax with and without chest tubes (Figure 2). The need for visual inspection of the images represents obvious limitations of large-scale data set generation, but since these models are increasingly available to the public, guidelines will be required to emphasize their limitations and direct their appropriate use.

A milestone in medical imaging data was achieved in 2022 with the publication of RadImageNet,^43^ an open radiologic deep learning data set that consists of 1.35 million radiologic images for efficient transfer learning. In our experimental evaluation of the reading race paper where we demonstrated superhuman ability for AI to predict self-reported race across multiple modalities, we found that some transfer learning tasks are better in race detection.^28^ The availability of a radiology specific data set to facilitate transfer learning is a step towards efficiency in model development. Such data set will also discourage the use of Frankenstein data sets—assembled from other data sets and redistributed under a new name—as pretraining can be done on RadImageNet and fine-tuned on a smaller data set. RadImageNet can and should learn from the pitfalls of its non-medical imaging counterpart, Imagenet, whose labels have had to be revised due to object co-occurrence, and whose distribution changed reflecting evolving patterns of Internet use over time.^44,45^

### Pitfalls in model development

Numerous articles have been published on algorithmic bias in medical imaging (during augmentation, modeling of loss functions, hyperparameter tuning and transfer learning) and thus this topic will not be a major focus of this article.^46–48^ In this section, we will discuss bias arising from demographic and clinical confounders, lessons from federated learning and the challenge of dealing with class imbalance and those associated with multimodal data sets. The greatest challenge of bias detection and mitigation remains the lack of a consensus definition of algorithmic bias despite many toolkits developed for this purpose. A model developer must determine, in consultation with clinicians, patients and communities what bias means for their model. For example, a developer will have to decide between aiming for group fairness (Black *vs* white patients, patients on government healthcare programs, or a specific age group) *vs* individual fairness^49^ (where two individuals with similar characteristics are treated in the same manner). This is further complicated by the fact that should a developer decide to aim for group fairness, then various approaches can be applied including ignoring any protected attribute like race (fairness through unawareness); or equalizing the proportions of various groups like an equal composition of images from Blacks and Whites (demographic parity)^50^; or the process of equalized odds or opportunity.^50^ Attempts to be race agnostic or race neutral are challenging because it remains difficult to remove race and race proxies like pattern of health service utilization because of patient and/or provider factors. In fact, these models have been shown to demonstrate the same bias patterns even when protective attributes are removed from the model variables.^1^

This is further complicated when models can pick up “hidden signals”—clinical, demographic or technology-related confounders—in the data sets that may not be obvious to the model developer. AI models learn the underlying data set distribution instead of disease characteristics leading to model failure.^51^ This phenomenon, also called shortcut learning, can be mitigated using various techniques including feature disentanglement (where you reward a model when it learns disease characteristics and penalize the model when it learns features that can discriminate between the original data sets where the images are obtained)^51^; applying pre-processing techniques like histogram equalization to correct for differences in the images and lung masking to exclude areas where pathology is not expected.^52^ Detecting these shortcuts and avoiding shortcut learning is an active area of research.

Federated learning—whereby models are trained across multiple institutions without the data being shared except for the model weights—has been proposed as a solution to improve model performance across diverse patient groups. In our own experience,^53^ we observed that pre-processing of images is important and affects the federated model performance. When we used the FedAvg algorithm, the model improved its generalizability on external data sets but at the expense of some degradation in internal validation. Moreover, FedAvg is biased to research sites contributing larger data sets with poor performance reported for smaller data sets. Overall, when local model performance matters—then personalized federated learning models (such as FedBN) should be prioritized, while for external performance, FedAvg should be used.^53^ It should be noted that model auditing and bias detection, which benefit from diverse perspectives during training and validation, are more difficult in federated learning where only the weights are shared across the groups of modelers.

Class imbalance occurs frequently in radiology. Radiology images tend to have high pixel dimensions and resolutions, yet pathology is represented on a small percentage of the pixels. For example, a screening mammogram has four views, yet the abnormality, *e.g.* a small cluster of calcifications, may appear on less than 3% of the image surface area. Beyond individual images, class imbalance also occurs due to distribution of positive versus negative cases (since most studies are normal), and across various demographic distributions within data sets. Several strategies are applied to mitigate this imbalance—including downsampling the majority class, upsampling the minority class, use of image augmentation techniques—and can change the training data set composition or cause model overfitting in small data sets with tightly curated features causing poor performance for the minority class. These techniques do not address sampling selection bias such as what in the example described earlier on Black and Hispanic patients less likely to survive an out-of-hospital cardiac arrest. The Blacks and Hispanic patients who survive an out-of-hospital cardiac arrest and are included in a data set may be different from those who died before reaching the hospital and are included in the database. Up- and downsampling may worsen bias. It is important to note that while we do not know the perfect composition of training and validation data sets to yield fair algorithms, sex imbalance in the data set has been demonstrated to have inferior performance for the minority group.^54^ The assumption that inclusion of more minority groups in the training data set is sufficient to fix bias is incorrect for the reasons above and since patients exist at the intersection of multiple group representation *e.g.* Black, Black Males/Females, Black Males/Females (young or old), Old Black Males/Females (with/without insurance).

There is increased development of fusion models that combine other data sources to medical imaging data (pathology, clinical tabular data, genomics among others).^55^ These models have a larger feature space used for training, which require feature selection early in the training process. Signals to detect race and other sensitive attributes may become even easier to learn during training. Ascertaining that these signals do not influence predictions and optimizations by AI becomes even more problematic.

### Pitfalls in model evaluation and validation

Model validation remains a barrier that limits incorporation of AI into clinical care. Many factors affect AI validation—including high costs of validation (technical pipeline for data set preparation and curation, diverse team of machine learning and clinical experts, regulatory approval burden), and lack of consensus on what is the proper protocol of validation. It is critical that appropriate metrics are identified and reported for the clinical task being performed. A review of 151 imaging AI products cleared by the FDA by November 2021 showed that only 64% of these products used clinical data for their validation; and these clinical data had limited information on demographic and technical confounders (only 4% had patient demographics and 5% reported machine specifications).^56^ Moreover, only 34% had multi-institutional validation and reported which institution(s) was(were) used for validation.^56^ It is thus not surprising that most of these algorithms, despite regulatory approval, demonstrate bias when deployed to various clinical settings especially when the target population is different from the population the model was trained on. In these cases, the AI models “fail silently” *i.e.* they do not provide an output to the user when they do not recognize the model input. These AI models will make a guess estimate of the prediction for wrong input data without providing the level of uncertainty. Differences in data distribution between model training and real-world deployment or external validation is also referred to the data being “out of distribution”.

External validation can ensure models generalize well across populations. Despite infrequent to even non-existent external validation for both FDA approved and peer reviewed articles, when external validation is performed, it is typically limited in scope. A recent systematic review found that 86 studies have a median of 240 cases used for external validation, with about 47% of which are positive cases.^57^ Even on what would be considered a small sample size for external validation, nearly half of the 86 studies reported a modest increase in external performance with nearly a quarter reporting a substantial decrease, reiterating on the challenge of validation.^57^

It is critical to note that regulatory approval at its current state does not guarantee model fairness. Bias evaluation and mitigation frequently occurs *post-hoc*, and because the approval process require resubmission when models are updated, there are no incentives to address algorithmic bias during the initial application.

The FDA, Canadian Agency for Drugs and Technology, European Union, and the UK code of conduct for data-driven healthcare technology only provide high-level guidance to promote fairness. For example, these organizations issue statements like “consider evaluation of bias”, without clear instructions on how the evaluation should be performed, and what consequences result from not performing such evaluation.^11^ A review of AI reporting guidelines noted that references to bias are either absent, made in passing or included in the guideline supplement. While some people have called for more clinical trials in validating AI, we must acknowledge the limitations of such trials including high costs, the fact that most trials never meet their enrollment proportions, and that bias still exists in clinical trials despite their rigor. In fact, a review of the use of clinical trials for machine learning in healthcare found only 41 randomized clinical trials, and most did not adhere to accepted reporting guidelines and had limited participation from minority groups.^58^ The regulatory landscape is changing as exemplified by the actions of four federal US agencies who recently issued a joint statement against biased models and their use including non-medical use like employment discrimination.^59^

Proprietary models present an additional layer of complexity because they are usually trained and validated on private data sets that are not accessible for other researchers to evaluate the model. This “black box inside a black box” nature of commercial AI models and concerns for protecting intellectual is recipe for AI perpetuating or even scaling health inequities.^60^ Evaluation of proprietary algorithms, when performed, involves simulations to try and replicate model performance. Academic-industry partnerships will be required to perform comprehensive model evaluation including bias. It is encouraging to note that the technology to support these types of experiments (including some that show portions of the data or blind the code to the evaluators) are being developed as part of trusted research environments.

### Pitfalls in model implementation and deployment

Despite obtaining the relevant regulatory approval, safely translating models from bench to bedside is even more challenging than algorithm development. Vendors must navigate the convoluted process of integrating with current hospital IT systems, and engaging clinicians and patients. Moreover, in many cases, the models do not automatically perform well out of the box and require some fine tuning and calibration to the local site data. This test data must be created by the local IT team and then curated carefully by the local clinicians who may not be familiar with the requisite subgroup model performance evaluation and other unintended consequences of AI bias.^61^ Due to lack of IT infrastructure and process for continuous model monitoring, changes in software or image acquisition equipment or protocols can result in model failure that may not be immediately detected, if at all. An interesting observation is that the type of explanation matters when it comes to biasing the end user. For example, the use of a machine learning algorithm by pathologists to differentiate different liver cancer types found that the pathologist accuracy did not improve when the model’s prediction was correct; however, the pathologist performance worsened when the model’s prediction was incorrect.^62^ Human–machine collaboration is now at the forefront of improving model performance, and to prevent unintended consequences including automation bias.

## Discussion

In this paper, we highlight bias that can occur at each stage of AI model development. Moreover, we frame this discussion in the context of known AI model pitfalls potential where medical imaging AI can fail. As highlighted in Figure 1, bias at each stage of model development can occur due to machine factors, human factors or a combination of both. Moreover, bias at one stage can be propagated to other stages, hence efforts to detect and mitigate bias should be at each stage. At each stage of the manuscript, we provide suggestions on how to avoid bias. In the section below, we recap on some broad principles for mitigating bias in AI for medical imaging.

First and foremost, we urge researchers and practitioners should avoid the exclusion of diverse and underrepresented populations when collecting and selecting training data. We also caution against broad grouping of underrepresented populations into the “Other” category which is common when researchers have small sample sizes. Neglecting continents and ethnically diverse locations perpetuates health inequities and hinders the growth and applicability of AI models. Similarly, relying on data sets with narrow geographic and other dimensions of diversity can further biases and limits generalizability of AI technologies. Reproducibility of results is another concern that must be addressed. Failure to provide transparent documentation and detailed methodologies especially for commercial algorithms has potential to perpetuate biases and prevent critical analysis. Following guidelines for research documentation and complete reporting increases transparency.^63,64^ We encourage researchers to ensure that their work is reproducible, allowing for independent validation and bias detection. We urge caution on relying on only mathematical approaches of fairness evaluation (*e.g.* relying solely on fairness through unawareness, demographic parity, or equalized odds or opportunity), as these approaches may overlook nuanced biases and fail to address systemic issues. The most challenging issue for bias in medical imaging arises from techniques to address class imbalance and how to evaluate and mitigate harmful “hidden signals” in imaging data sets, as they can lead to shortcut learning and reinforce existing biases.

To mitigate bias, we must continue to create and use diverse and representative data sets, develop and test rigorous testing and validation protocols, perform ongoing monitoring and evaluation of model performance. Bias risk assessment tools can facilitate this process.^65^ Bias mitigation is especially important as we understand human–machine partnership, with early findings showing worsening performance for experts when presented with biased model outputs. Most importantly, we cannot overemphasize the need for diverse teams to work on this challenging topic. By taking a comprehensive and multifaceted approach to addressing bias in AI model development, researchers and practitioners can help to ensure that these technologies are used ethically and responsibly to benefit all patients.

## Conclusion

Bias occurs across the AI life cycle, and hence must be tackled at each step from problem definition all the way through post-deployment monitoring. Radiology AI performance is affected by other underlying practice patterns which can appear as hidden signals in AI data sets. Due to limited AI explainability, these hidden signals can be difficult to detect and have led to algorithmic bias that encrypts health inequities into practice. Ensuring that AI works for all will require diverse teams in addition to diverse data sets.
